# Tumor Treating Fields (TTFields) increase the effectiveness of temozolomide and lomustine in glioblastoma cell lines

**DOI:** 10.1007/s11060-023-04308-4

**Published:** 2023-05-02

**Authors:** Hila Fishman, Roni Monin, Eyal Dor-On, Adrian Kinzel, Adi Haber, Moshe Giladi, Uri Weinberg, Yoram Palti

**Affiliations:** 1 Novocure Ltd, Haifa, Israel; 2 Novocure, GmbH, Munich, Germany

**Keywords:** Glioblastoma, Tumor Treating Fields (TTFields), Temozolomide, Lomustine, DNA damage

## Abstract

**Purpose:**

Tumor Treating Fields (TTFields) are electric fields that disrupt cellular processes critical for cancer cell viability and tumor progression, ultimately leading to cell death. TTFields therapy is approved for treatment of newly-diagnosed glioblastoma (GBM) concurrent with maintenance temozolomide (TMZ). Recently, the benefit of TMZ in combination with lomustine (CCNU) was demonstrated in patients with O^6^-methylguanine DNA methyltransferase (MGMT) promoter methylation. The addition of adjuvant TTFields to TMZ plus CCNU further improved patient outcomes, leading to a CE mark for this regimen. The current in vitro study aimed to elucidate the mechanism underlying the benefit of this treatment protocol.

**Methods:**

Human GBM cell lines with different *MGMT* promoter methylation statuses were treated with TTFields, TMZ, and CCNU, and effectiveness was tested by cell count, apoptosis, colony formation, and DNA damage measurements. Expression levels of relevant DNA-repair proteins were examined by western blot analysis.

**Results:**

TTFields concomitant with TMZ displayed an additive effect, irrespective of MGMT expression levels. TTFields concomitant with CCNU or with CCNU plus TMZ was additive in MGMT-expressing cells and synergistic in MGMT-non-expressing cells. TTFields downregulated the FA-BRCA pathway and increased DNA damage induced by the chemotherapy combination.

**Conclusions:**

The results support the clinical benefit demonstrated for TTFields concomitant with TMZ plus CCNU. Since the FA-BRCA pathway is required for repair of DNA cross-links induced by CCNU in the absence of MGMT, the synergy demonstrated in *MGMT* promoter methylated cells when TTFields and CCNU were co-applied may be attributed to the BRCAness state induced by TTFields.

**Supplementary Information:**

The online version contains supplementary material available at 10.1007/s11060-023-04308-4.

## Introduction

Glioblastoma (GBM), a grade 4 glioma, is the most common malignant primary brain tumor. In 2005, the Stupp protocol was established for treatment of newly-diagnosed GBM, consisting of maximal safe tumor resection, followed by radiation with concomitant and adjuvant treatment with the chemotherapy temozolomide (TMZ) [1]. Tumor Treating Fields (TTFields), electric fields that disrupt cellular processes critical for cancer cell viability and tumor progression [2–4], were approved by the United States Food and Drug Administration (FDA) in 2015 for concomitant use with maintenance TMZ in newly-diagnosed GBM patients, followed by approvals in several European Union countries, as well as Switzerland, China, Japan, Canada, Hong Kong, Australia, and Israel [5, 6].

Promoter methylation of O^6^-methylguanine-DNA methyltransferase (MGMT)—the major enzyme involved in repair of DNA damage induced by TMZ [7–10]—serves as a useful predictor for glioma responsiveness to TMZ [11–13]. Promoter methylation of the *MGMT* gene prevents its expression, leading to better outcomes in response to TMZ administration [14, 15]. Unfortunately, many GBM tumors have an unmethylated *MGMT* promoter, and decreased promoter methylation may occur upon tumor progression or recurrence, leading to TMZ-resistance [7–9].

The acquired resistance to TMZ makes this drug less effective in the recurrent setting. Other treatments approved for recurrent GBM include [16, 17]: the alkylating agents lomustine (CCNU) and carmustine (BCNU), which—unlike TMZ—may induce DNA cross-linking downstream of the preliminary guanine alkylation [10, 18]; bevacizumab, an antibody that binds and inhibits vascular endothelial growth factor (VEGF) thus preventing tumor angiogenesis; and TTFields [19].

Recently the CeTeG/NOA-09 trial demonstrated the advantage of CCNU plus TMZ in newly-diagnosed GBM patients with a methylated *MGMT* promoter [20]. In light of these results, a bicentric retrospective analysis was performed on data from patients with newly-diagnosed GBM and a methylated *MGMT* promoter who were treated with TTFields, TMZ, and CCNU after completing chemoradiation [21]. The study reported the safety, feasibility, and initial efficacy of this treatment protocol. A recent multi-center analysis of real-world evidence corroborated those findings, demonstrating a survival benefit for TTFields with TMZ plus CCNU [22]. These studies were the basis for expanding the CE Mark to include CCNU concomitant with TTFields and maintenance TMZ for newly diagnosed GBM patients.

The aim of the current study was to examine the underlying mechanism of action for TTFields with TMZ and CCNU, by comparing MGMT methylated and unmethylated GBM cell lines.

## Materials and methods

### Cell culture

Human GBM cell lines U-87 MG, LN-229, U-118 MG, and LN-18 were obtained from the American Tissue Culture Collection (ATCC). Cells were grown in DMEM media supplemented with 5 or 10% (v/v) fetal bovine serum (FBS), 2 mM L-glutamine and penicillin/streptomycin (50 µg/ml) in a 37 °C humidified incubator supplied with 5% CO_2_. Media and supplements were purchased from Biological Industries Ltd. (Beit Haemek). U-87 MG and U-118 MG TMZ-resistant cell lines were generated from parental cell lines by culturing with 100 µM TMZ for two months, with daily replacements for five days and then every three days.

### TTFields-chemotherapy co-application experiments

The inovitro™ system (Novocure, Haifa, Israel) was utilized for applying TTFields to the cells (0.83 V/cm RMS, 72 h), at the GBM optimal 200 kHz frequency, as previously described [23–25]. For chemotherapy dose–response curves, 5–1000 µM TMZ (Sigma-Aldrich T2577) or 8–125 µM CCNU (Sigma-Aldrich L5918) were applied, with or without TTFields. For testing TTFields together with TMZ and CCNU, the following chemotherapy concentrations were used: 25 µM TMZ and 8.7 µM CCNU for U-87 MG cells; 12 µM TMZ and 5 µM CCNU for LN-229 cells; 30 µM TMZ and 10 µM CCNU for U-118 MG cells; and 100 µM TMZ and 15 µM CCNU for LN-18 cells.

### Cell count

Cell number was determined by cell counting using iCyt EC800 flow cytometer (Sony Biotechnology, San Jose, CA, USA). Results are presented as a percentage of control.

### Apoptosis

For apoptosis analysis, cells were stained with FITC-conjugated Annexin V (AnnV) and 7-Aminoactinomycin D (7-AAD) using a commercial kit (BioLegend, San Diego, CA, USA). Data acquisition and analysis were done on the iCyt EC800 flow cytometer (Sony Biotechnology, San Jose, CA, USA).

### Clonogenicity and overall effect

Cells were harvested, re-plated (500 cells/well, 6-well plates), and grown for 21 days. Colonies were quantified with ImageJ after 0.5% crystal violet staining and expressed as percentages of control. Overall effect was calculated by multiplying cell count and the corresponding clonogenic effect.

### DNA damage examination

Cells were fixed for 10 min with 4% paraformaldehyde, permeabilized with 0.5% Triton X-100 in PBS for 20 min and blocked with donkey serum (PBS with 0.3% triton, donkey serum 1:100). Cells were then stained overnight at 4 °C with anti-ɣH2AX antibody (Cell Signaling, Danvers, MA, USA; #9718, 1:400), followed by incubation for 1 h at room temperature with Alexa Flour 488-conjugated secondary antibody (Jackson Immunoresearch, Cambridge, UK; #711–545-152, 1:500) and 0.2 μg/ml 4′,6-diamidino-2-phenylindole (DAPI; Sigma Aldrich, Rehovot, Israel). Images were collected using LSM 700 laser scanning confocal system (Zeiss, Gottingen, Germany). Mean number of foci per nucleus was determined using the FIJI software with the BioVoxxel plugin.

### Western blot analysis

Cell extracts were prepared and subjected to western blot analysis (40 μg protein/sample), using primary antibodies from Table 1 followed by incubation with horseradish peroxidase (HRP)-conjugated secondary antibody (Abcam, Cambridge, UK; #ab97023 or #ab6721, 1:10,000). A chemiluminescent substrate (Immobilon Forte, Millipore, Burlington, MA, USA) was used for visualization, and signals were recorded on GeneGnome XRQ gel imager (AlphMetrix Bitech, Rödermark, Germany). Densitometric readings were normalized to GAPDH with FIJI software and expressed as fold change relative to control.

**Table 1:** Primary antibodies used in the study for Western Blot analysis

Antigen	Vendor	Catalog Number	Dilution
BRCA2	Cell signaling	10741	1:1000
FANCB	Cell signaling	14243	1:1000
FANCD2	Cell signaling	16323	1:1000
FANCJ	Cell signaling	4578	1:1000
GAPDH	Santa cruz	SC-32233	1:2000

### Statistical analysis

Experiments were repeated at least three times, and data are presented as mean ± standard error of the mean (SEM). Statistical significance was calculated using GraphPad Prism 8 software (La Jolla) and differences considered significant at values of: *p < 0.05, **p < 0.01, ***p < 0.001, and ****p < 0.0001.

## Results

### Effectiveness of TTFields in GBM cell lines is independent of MGMT expression level or TMZ resistance

We first characterized MGMT expression levels and TMZ sensitivity of the four GBM cell lines selected for the study. In accordance with their previously reported promoter methylation status [26, 27], MGMT protein expression was not detected in the *MGMT* promoter methylated  U-87 MG and LN-229 cells. MGMT was however expressed in the U-118 MG cells with a partially methylated *MGMT* promoter and in the LN-18 cells with an unmethylated *MGMT* promoter, though to a much higher extent in the latter (Fig. S1A). Sensitivity to the cytotoxic effect of TMZ correlated with MGMT expression levels, with U-87 MG and LN-229 cells being the most sensitive, and the LN-18 cell line being the least sensitive (Fig. S1B).

**Fig. 1:**
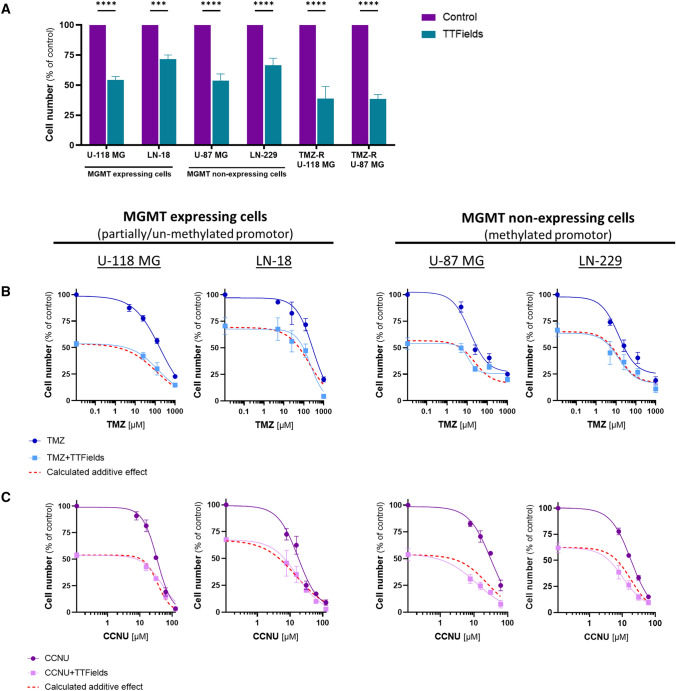
Effectiveness of TTFields in MGMT-expressing and MGMT-non-expressing GBM cell lines, with or without TMZ or CCNU. Cell count following application of TTFields (200 kHz, 0.83 V/cm, 72 h) to human GBM cell lines: MGMT-expressing U-118 MG and LN-18 cells; MGMT-non-expressing U-87 MG and LN-229 cells; and TMZ-resistant (TMZ-R) U-118 MG and U-87 MG cells, generated by repeated exposure to high-dose TMZ (**A**). Values are mean ± SEM. ***p < 0.001, and ****p < 0.0001 relative to control; 2-way ANOVA, Sidak’s multiple comparison. Cell count following 72 h cell treatment with various doses of TMZ (**B**) or CCNU (**C**) alone (dark blue and purple lines, respectively) or concomitant with TTFields (200 kHz, 0.83 V/cm RMS; light blue and purple lines, respectively). Values are mean ± SEM. The calculated additive effects are depicted as red dashed lines. In all cell lines, *p* was < 0.001 for the dose effect of both chemotherapies and for the effect of TTFields; 2-way ANOVA

Despite the differences between the four cell lines in MGMT expression levels and TMZ sensitivity, they all exhibited a comparable response to relatively low intensities of TTFields (0.83 V/cm), demonstrating a significant reduction in cell count (53%–71% of control) (Fig. 1A).

As resistance to TMZ may stem from other factors apart from MGMT expression levels [9], we next generated TMZ-resistant cells by long-term exposure of U-87 MG and U-118 MG cells to TMZ. These TMZ-resistant cells demonstrated profoundly lower sensitivity to TMZ relative to the parental cells, which was not mediated by differences in MGMT expression levels (Fig. S2); nevertheless, sensitivity of these cells to TTFields was retained (Fig. 1A).

### TTFields additively enhance the effectiveness of TMZ in GBM cell lines, irrespective of MGMT expression levels

Next, we examined the effect of concomitant exposure to TTFields and TMZ in the four parental cell lines (Fig. 1B). The dose dependent response of the cells to TMZ administration (dark blue lines) was preserved in the presence of TTFields (light blue lines) (*p* < 0.001), with TTFields augmenting the effect induced by TMZ alone (*p* < 0.001).

To elucidate the nature of the TTFields-TMZ interaction, the expected magnitude for an additive effect was calculated by multiplying the corresponding values for the independent treatments. The overlap of these calculated curves (Fig. 1B**,** red dashed lines) with the measured TTFields plus TMZ curves indicated an additive interaction between the two modalities in all examined cell lines, irrespective of MGMT expression levels.

### TTFields enhance the effectiveness of CCNU, additively in MGMT-expressing and synergistically in MGMT-non-expressing GBM cells

The four cell lines demonstrated comparable sensitivities to CCNU (Fig. S3). The dose dependent response of the cells to administration of CCNU (dark purple lines) was retained when TTFields were co-applied (light purple lines) (*p* < 0.001), with TTFields amplifying the effect induced by CCNU alone (*p* < 0.001) (Fig. 1C).

Examination of the type of interaction between TTFields and CCNU (Fig. 1C, red dashed lines) revealed an additive interaction in the MGMT-expressing U-118 MG and LN-18 cells (measured and calculated curves overlapping), and a synergistic interaction in the MGMT-non-expressing U-87 MG and LN-229 cells (measured curves below calculated curves).

### TTFields enhance the effectiveness of TMZ + CCNU in GBM cell lines, with higher benefit in MGMT non-expressing cells

**Fig. 2:**
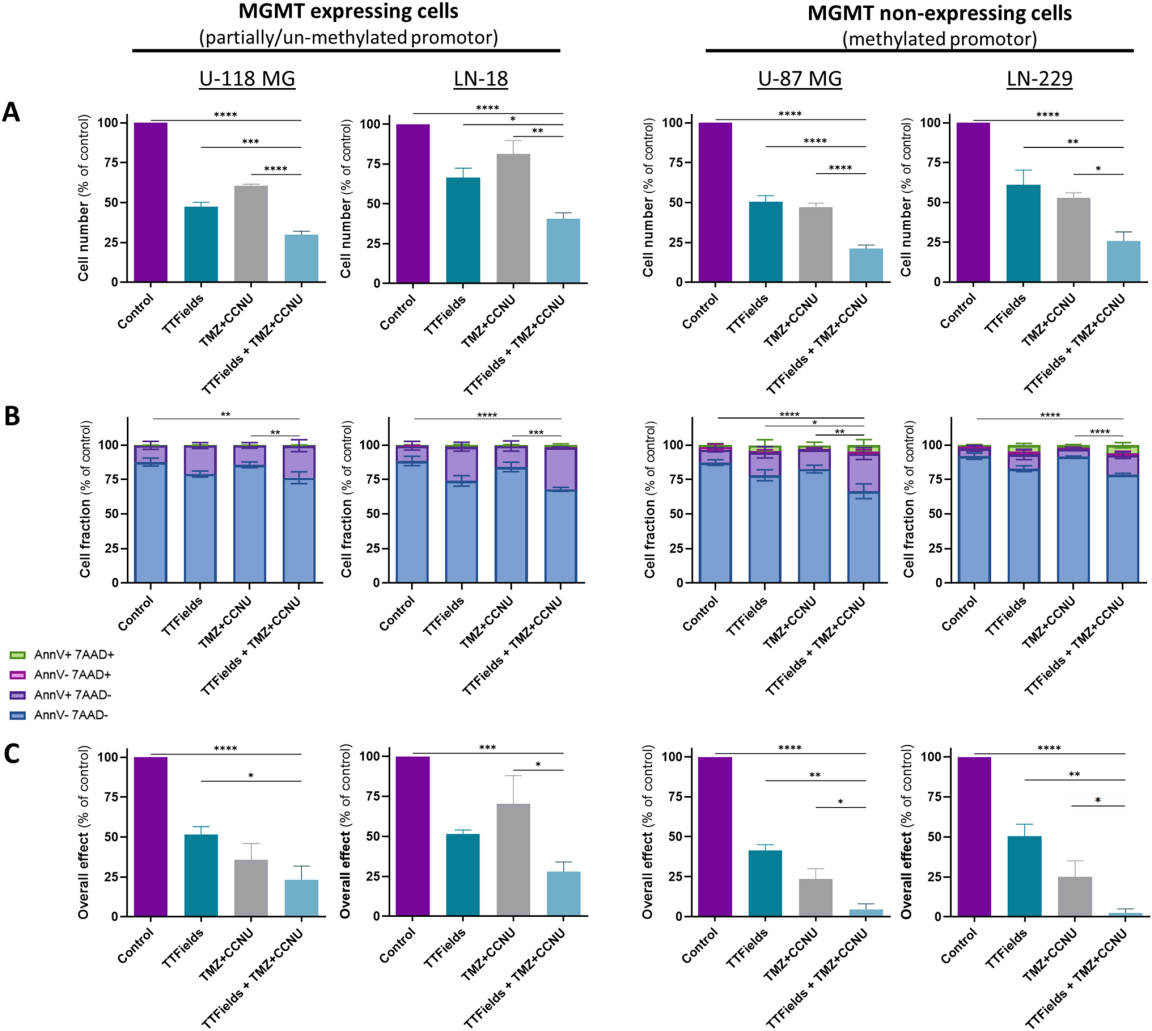
Effectiveness of TTFields in MGMT-expressing and MGMT-non-expressing GBM cell lines, with or without TMZ + CCNU. MGMT-expressing U-118 MG and LN-18 cells, and MGMT-non-expressing U-87 MG and LN-229 cells were treated for 72 h with TMZ + CCNU, TTFields (200 kHz, 0.83 V/cm RMS), or both treatments together, followed by examination of cell count (**A**), apoptosis (**B**), and overall effect (**C**). Chemotherapy doses: 30 µM TMZ and 10 µM CCNU for U-118 MG cells; 100 µM TMZ and 15 µM CCNU for LN-18 cells; 25 µM TMZ and 8.7 µM CCNU for U-87 MG cells; and 12 µM TMZ and 5 µM CCNU for LN-229 cells. Values are mean ± SEM. *p < 0.05, **p < 0.01, ***p < 0.001, and ****p < 0.0001 relative to TTFields + TMZ + CCNU; Dunnett’s multiple comparisons

Next, we examined the effect of TTFields application in the presence of both TMZ and CCNU. In the various cell lines, TTFields alone reduced cell count to 47%–67% of control, and a similar decrease to 47%–81% of control was seen for the TMZ + CCNU combined treatment (Fig. 2A). Concomitant TTFields with TMZ + CCNU application significantly reduced cell count compared to each modality alone, leading to cell count of 21%–41% relative to control.

To test whether the effect on cell count was the result of proliferation arrest or actual cell death, we examined the cells by an apoptosis assay at treatment cessation (Fig. 2B). Control cells presented with 87%–92% live cells (AnnV-7AAD- cells) in the various cell lines, TTFields lowered the percentage of live cells to 74%–83%, while TMZ + CCNU treatment had no significant effect (83%–92% live cells). When TTFields were applied together with TMZ + CCNU, live cell percentage was further reduced to 66%–79%, which was significantly lower relative to untreated and TMZ + CCNU treated cells.

At treatment end, we also examined the ability of the surviving cells to proliferate further; the clonogenic capacity was then combined with the corresponding cell count to evaluate the overall effect (Fig. 2C). TTFields enhanced the overall effect of TMZ + CCNU in all examined cell lines, however this effect did not reach statistical significance in the U-118 MG cells do to the relatively high variance in the clonogenicity of this cell line. The interaction of TTFields with the chemotherapies was revealed to be additive in MGMT-expressing cells (U-118 MG—19.6% expected for additivity versus 23.3% observed; LN-18—34.0% expected for additivity versus 28.0% observed) and synergistic in MGMT-non-expressing cells (U-87 MG—9.98% expected for additivity versus 4.50% observed; LN-229—13.4% expected for additivity versus 2.50% observed).

### TTFields increase DNA damage induced by TMZ + CCNU and downregulate the Fanconi Anemia-BRCA pathway in GBM cell lines

**Fig. 3:**
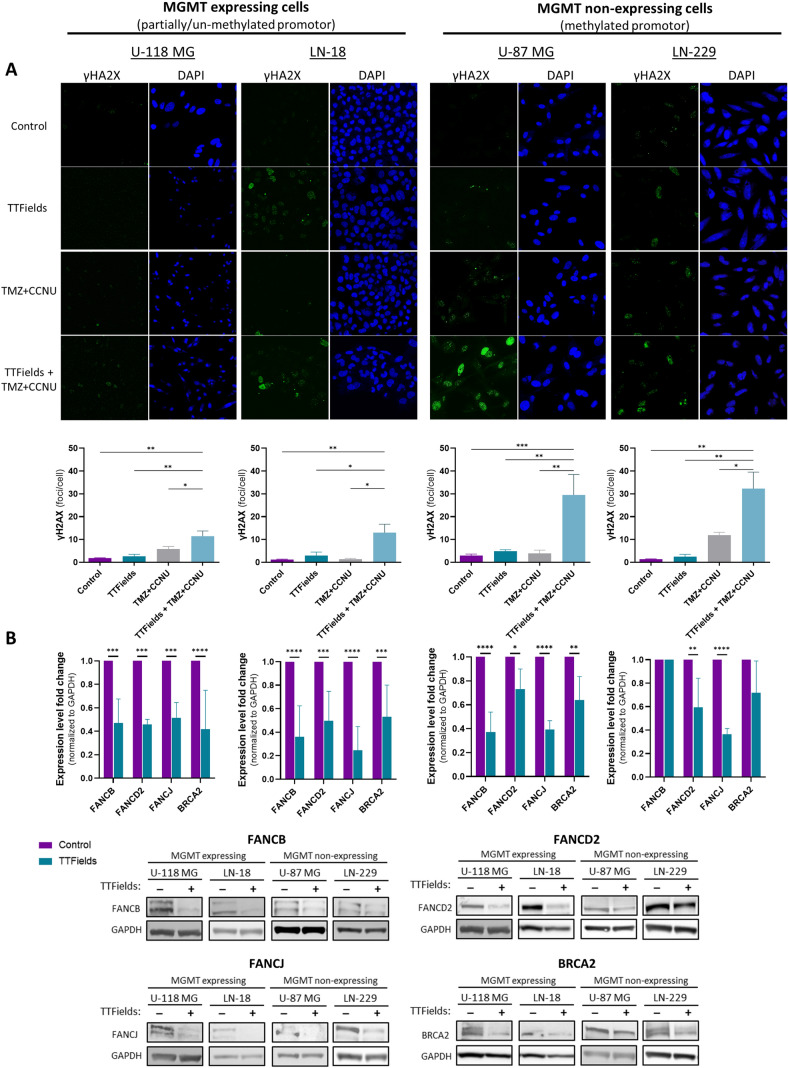
Effects of TTFields on TMZ + CCNU induced DNA damage and on the FA-BRCA DNA repair pathway in GBM cells. **A** MGMT-expressing U-118 MG and LN-18 cells, and MGMT-non-expressing U-87 MG and LN-229 cells were treated for 72 h with TMZ + CCNU, TTFields (200 kHz, 0.83 V/cm RMS), or both treatments together, followed by immunofluorescence staining of ɣH2AX (green) for detection of DNA damage and DAPI (blue) for nuclear visualization. Presented are representative images at × 20 magnification, and quantification of mean foci per nucleus. Chemotherapy doses: 30 µM TMZ and 10 µM CCNU for U-118 MG cells; 100 µM TMZ and 15 µM CCNU for LN-18 cells; 25 µM TMZ and 8.7 µM CCNU for U-87 MG cells; and 12 µM TMZ and 5 µM CCNU for LN-229 cells. Values are mean ± SEM. *p < 0.05 and **p < 0.01 relative to TTFields + TMZ + CCNU; Dunnett’s multiple comparisons. **B** Cells were treated for 72 h with TTFields (200 kHz, 0.83 V/cm RMS), followed by immunoblotting of cell lysates for expression of FANCB, FANCD2, FANCJ, and BRCA2. Values are mean ± SEM. *p < 0.05, **p < 0.01, ***p < 0.001, and ****p < 0.0001 relative to control; 2-way ANOVA, Sidak’s multiple comparison

We further examined treated cells by fluorescence microscopy, staining the cells with DAPI for nuclear visualization, and with an anti-γH2AX antibody for assessment of DNA damage (Fig. 3A). The control cells presented with a basal level of 1 to 3 γH2AX foci/nucleus, which was slightly elevated following treatment with TTFields or TMZ + CCNU alone, to a level of 2 to 6 foci/nucleus (excluding the case of TMZ + CCNU in LN-229 cells, for which 12 foci/nucleus were seen).

When TTFields were applied together with TMZ + CCNU (Fig. 3A), about 12 foci/nucleus were observed in the MGMT-expressing U-118 MG and LN-18 cells, while a substantial elevation to about 30 foci/nucleus was detected in the MGMT-non-expressing U-87 MG and LN-229 cells. Overall, concurrent TTFields with TMZ + CCNU was more effective than the individual treatments in inducing DNA damage, with this effect more profound in cell lines not expressing MGMT.

To explore this elevated DNA damage, we examined possible changes in expression levels of proteins involved in DNA damage repair in response to TTFields application. Specifically, expression of proteins from the Fanconi Anemia (FA)-BRCA pathway was evaluated, as TTFields have been shown to downregulate this pathway in cells from other tumor types [28, 29]. Indeed, TTFields application decreased the expression of FANCB, FANCD2, FANCJ and BRCA2 relative to untreated cells (Fig. 3B).

## Discussion

The primary aim of this work was to study in cell cultures the effects of adding TTFields to the CeTeG regimen, a strategy with a recently identified potential clinical benefit [21, 22]. The work described herein focused on the involvement of TTFields in DNA damage and repair as the other modalities used for treatment of GBM involve generation of DNA damage. It is however important to mention that TTFields induce additional effects on cancer cells, including the established antimitotic effect, which may also contribute to the effectiveness of this treatment. As TTFields are a physical treatment modality, and as electrostatic interactions are involved in multiple physiological and pathological processes, this multi-mechanistical nature of TTFields is not surprising, and may also involve other yet to be identified mechanisms.

Since GBM sensitivity to standard-of-care TMZ is greatly dependent on *MGMT* promoter methylation status [11–13], and may also be hindered by other mechanisms [9], we first examined whether sensitivity to TTFields may also depend on MGMT expression level or on MGMT-independent cellular TMZ-resistance. All tested GBM cell lines, both expressing and non-expressing MGMT, demonstrated comparable sensitivity to TTFields, similar to previously reported results [30, 31]. Furthermore, MGMT expressing and non-expressing TMZ-resistant cells, in which MGMT-independent resistance was generated by repeated exposure to high dose TMZ, exhibited comparable sensitivity to TTFields as the parental cell lines. Overall, the results show that sensitivity to TTFields was irrespective of the MGMT expression level and unrelated to the TMZ-sensitivity of the cells, in line with TTFields and TMZ having different, non-overlapping mechanisms of action.

Next, we examined the effects of TTFields when applied together with TMZ or CCNU. The potential benefit of adding TTFields to TMZ was additive, with no dependence on cellular MGMT expression status. An additive interaction was also seen between TTFields and CCNU in the MGMT-expressing cells, while a tendency to synergism was displayed in the MGMT-non-expressing cells. These in vitro results suggest potential benefit for concomitant application of TTFields with CCNU in recurrent GBM (in which *MGMT* promotor methylation is common), for which treatment options today encompass each of these modalities alone. While cellular sensitivity to TTFields or CCNU was independent of MGMT expression, their concurrent effect was dependent on expression of this enzyme, suggesting a crosstalk between the mechanism of action of the two modalities manifested only when they are co-applied.

Testing the concomitant application of TTFields with TMZ + CCNU relative to TTFields or TMZ + CCNU alone revealed higher reduction in cell count, elevated apoptosis, and enhanced overall effect, indicative of greater cytotoxicity and increased inhibition of post-treatment proliferation for the concomitant treatment. These effectivity measures revealed an additive interaction between TTFields and the chemotherapy combination in the MGMT-expressing cells and synergism in the MGMT-non-expressing cells. As a similar type of interaction was revealed in the experiments testing TTFields with CCNU alone, we speculate that the observed synergy in the case of TTFields with TMZ + CCNU derives from the suggested interaction between the mechanisms of action of TTFields and CCNU.

Since TTFields (in addition to and independent of their anti-mitotic effects) have been shown to impair DNA damage repair and promote accumulation of DNA double strand breaks in non-small cell lung cancer (NSCLC) and pleural mesothelioma cell lines [28, 29, 32], and as TMZ and CCNU are DNA alkylating agents, we next examined the effect of these modalities on DNA damage and repair in GBM cells. Indeed, DNA damage was elevated when TTFields were co-applied with TMZ + CCNU relative to each treatment alone; and as in the case of the efficacy tests, the effect was more pronounced in the absence of MGMT. TTFields application also reduced expression of proteins from the FA-BRCA pathway, demonstrated for FANCB of the core complex, FANCD2 from the ID complex, and for two pivotal downstream proteins FANCJ and BRCA2. While this effect was seen in MGMT expressing and non-expressing GBM cells alike, the manifestation of the effect—additive versus synergistic interaction with CCNU—was dependent on the MGMT expression status.

Previous studies in other tumor types have shown that TTFields downregulate the FA-BRCA proteins at the RNA and protein levels, and that DNA damage induced by TTFields is associated with cell cycle arrest and upregulation of proteins involved in DNA damage response (p21 and p27) [28, 29, 32]. While induction of DNA damage and impairment of DNA damage repair following application of TTFields have been established across several different tumor types, the exact mechanism by which the electric fields mediate these effects is not yet fully understood.

**Fig. 4:**
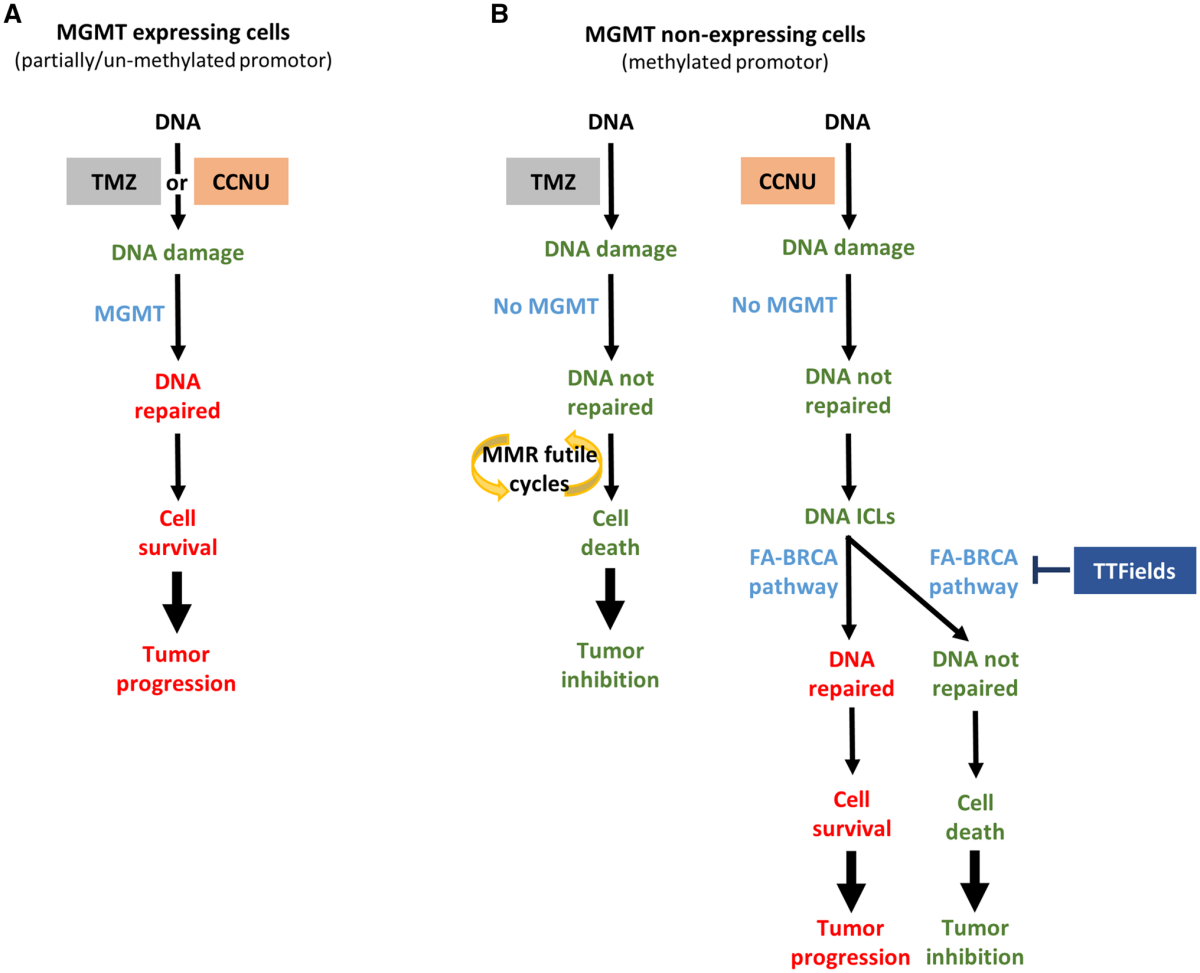
Mechanism of TMZ and CCNU induced cancer cell death, in MGMT-expressing and MGMT-non-expressing cells. **A** In MGMT-expressing GBM cells, cytotoxic O^6^ guanine alkylation damage induced by TMZ or CCNU is repaired by MGMT, allowing cell survival and tumor progression. **B** In MGMT-non-expressing GBM cells, the cytotoxic alkylation damage to O^6^ guanine is not repaired. The MMR pathway attempts to repair the TMZ-induced damage with no successes, resulting in cell death, while the FA-BRCA pathway successfully repairs the interstrand cross links (ICLs) formed downstream to the original CCNU-induced damage, allowing tumor progression. Application of TTFields induces a BRCAness state, inhibiting repair of ICLs formed by CCNU, and promoting cancer cell death

DNA damage is repaired through a variety of pathways, depending on the damage type. Alkylating agents damage DNA in normal and cancer cells, with their tumor-specific killing effect depending on cell proliferation rate and downregulation of DNA damage repair pathways in the cancerous cells. TMZ and CCNU both induce several types of DNA lesions, with the alkylation of the O^6^ position on guanine bases found to be most cytotoxic [8–10, 18]. While the more prevalent N-alkylations are mainly repaired by the base excision repair (BER) pathway, guanine O^6^-alkylation repair requires the MGMT enzyme. MGMT existence in normal cells is important for their protection, but when present in cancer cells it counter-acts the anti-cancer activity of the alkylating agents, conferring resistance in *MGMT* promoter unmethylated and partially methylated cells (Fig. 4A).

When MGMT is absent—as in cells with a methylated promoter—different repair pathways come into play for the two alkylating agents of interest (Fig. 4B). In the case of TMZ, the mismatch repair (MMR) machinery attempts to repair the damage with no success, resulting in cancer cell death and subsequent tumor decline [7–10]. However, unlike for TMZ, the primary damage induced by CCNU may evolve into interstrand crosslinks (ICLs), a severe form of DNA damage that can lead to cell death if left unrepaired [10, 18]. ICLs can be resolved by the FA-BRCA pathway to allow the unwanted rescue of the cancer cells and continued tumor growth [33, 34]. By downregulating the FA-BRCA pathway, TTFields may inhibit repair of ICLs, tilting the scale towards cell death and tumor decline. A similar synergistic interaction has previously been shown for application of TTFields together with cisplatin, another DNA damaging agent that induces ICLs [29].

Overall, the differences in the mechanisms by which each of the alkylating agents induce DNA damage, and the different measures employed by the cells to fix this damage, may account for the different types of interactions between TTFields and each of the chemotherapies. As the damage induced by TMZ does not require the FA-BRCA pathway for its repair, TTFields and TMZ each act independently to induce cell death, resulting in an additive interaction that is not affected by MGMT expression status; and the same rational holds true for the interaction of TTFields with CCNU in MGMT-expressing cells. While the individual effects of TTFields and CCNU were not dependent on MGMT expression status, the synergy seen between these two modalities in MGMT-non-expressing cells may be attributed to the need of the FA-BRCA pathway for repair of damage induced by CCNU in the absence of MGMT and the state of BRCAness induced by TTFields.

This BRCAness state induced by TTFields can support the previously demonstrated preclinical benefit of concomitant TTFields with radiation, another cancer treatment modality that induces DNA damage [32]. The potential use of TTFields therapy together with radiation therapy in patients with newly-diagnosed GBM is currently under clinical investigation in the TRIDENT trial (NCT04471844). The induced BRCAness state may also be exploited for use together with cancer treatments that inhibit other pathways of DNA damage repair, such as PARP inhibitors. As inhibition of PARP demonstrated effectiveness in patients with solid tumors and deleterious BRCA mutations [35], there may exist a potential benefit for TTFields together with PARP inhibitors in patients with wild type BRCA genes.

A limitation to the work we describe in this paper is that we utilized cell lines with different MGMT expression levels, as opposed to directly manipulating expression levels of this enzyme in selected cell lines (using pharmacological inhibitors or gene silencing approaches), hence providing evidence for synergy by association. A drawback for such an approach is that the cell lines examined differ genetically from each other in more than just MGMT expression, which was actually the rational for selecting this approach, as it better resembles the situation with real-life tumors. Importantly, the data described here is consistent with previous evidence for synergy of TTFields concomitant with other DNA damaging modalities that require the FA-BRCA pathway for repair.

In conclusion, DNA repair pathways are redundant and have back-up systems, allowing cancer cells to overcome stress by bypassing impaired pathways. Cancer treatments modulating DNA repair pathways may hence provide a promising strategy to increase effectiveness of chemotherapeutic alkylating agents. As such, a state of BRCAness induced by TTFields may be exploited to increase the effectiveness of agents that mediate DNA cross-linking, such as CCNU. Specifically, TTFields-induced downregulation of the FA-BRCA pathway promotes a synergistic interaction between TTFields and CCNU in *MGMT* promoter methylated GBM cells, and underscore the previously described clinical benefit of adding TTFields therapy to the CeTeG regimen for patients with a methylated *MGMT* promoter. A funding declaration is mandatory for publication in this journal. Please confirm that this declaration is accurate, or provide an alternative.

## Supplementary Information

Below is the link to the electronic supplementary material.

Supplementary file1 (PPTX 12669 KB)

## Declarations

### Data availability

All data generated or analyzed during this study are included in this article. Further inquiries can be directed to the corresponding authors.
