# Supplementary material for: Tumor Treating Fields (TTFields) increase the effectiveness of temozolomide and lomustine in glioblastoma cell lines
Source: J Neurooncol. 2023 May 2;163(1):83–94. doi: 10.1007/s11060-023-04308-4 (PMC10232578; doi:10.1007/s11060-023-04308-4)
Supplement: Supplementary file 1 — Supplementary file1 (PPTX 12669 KB) [file 11060_2023_4308_MOESM1_ESM.pptx]

## Slide 1
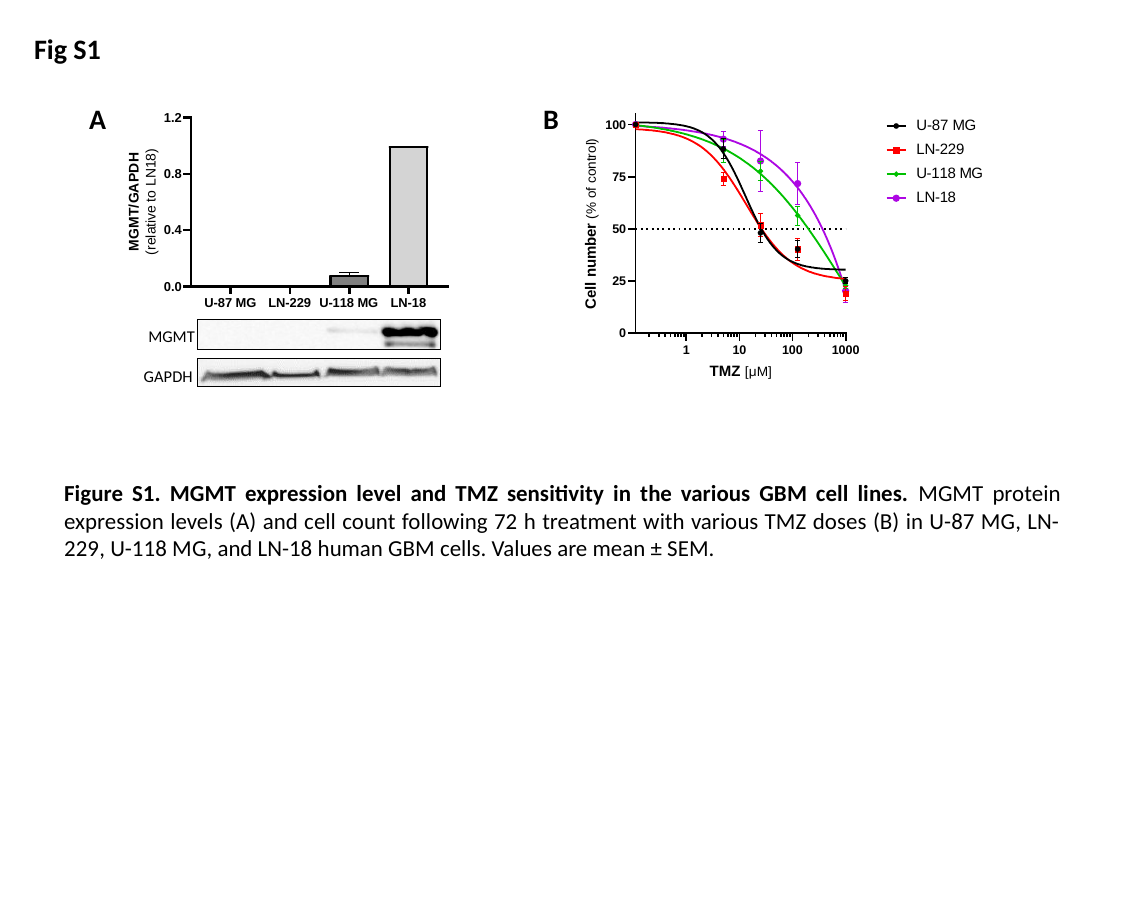

Fig S1
A
B
MGMT
GAPDH
Figure S1. MGMT expression level and TMZ sensitivity in the various GBM cell lines. MGMT protein expression levels (A) and cell count following 72 h treatment with various TMZ doses (B) in U-87 MG, LN-229, U-118 MG, and LN-18 human GBM cells. Values are mean ± SEM.

## Slide 2
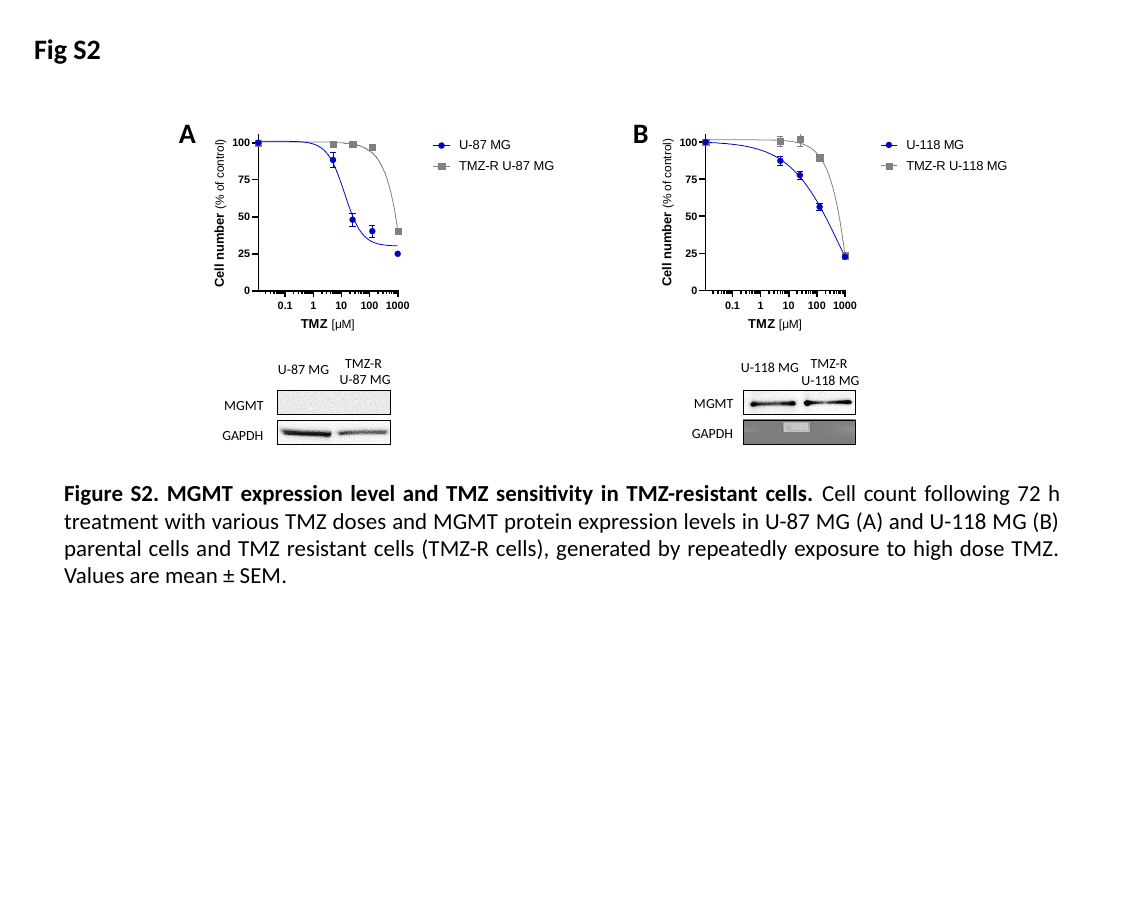

Fig S2
A
B
TMZ-R
U-87 MG
U-87 MG
MGMT
GAPDH
TMZ-R
U-118 MG
U-118 MG
MGMT
GAPDH
Figure S2. MGMT expression level and TMZ sensitivity in TMZ-resistant cells. Cell count following 72 h treatment with various TMZ doses and MGMT protein expression levels in U-87 MG (A) and U-118 MG (B) parental cells and TMZ resistant cells (TMZ-R cells), generated by repeatedly exposure to high dose TMZ. Values are mean ± SEM.

## Slide 3
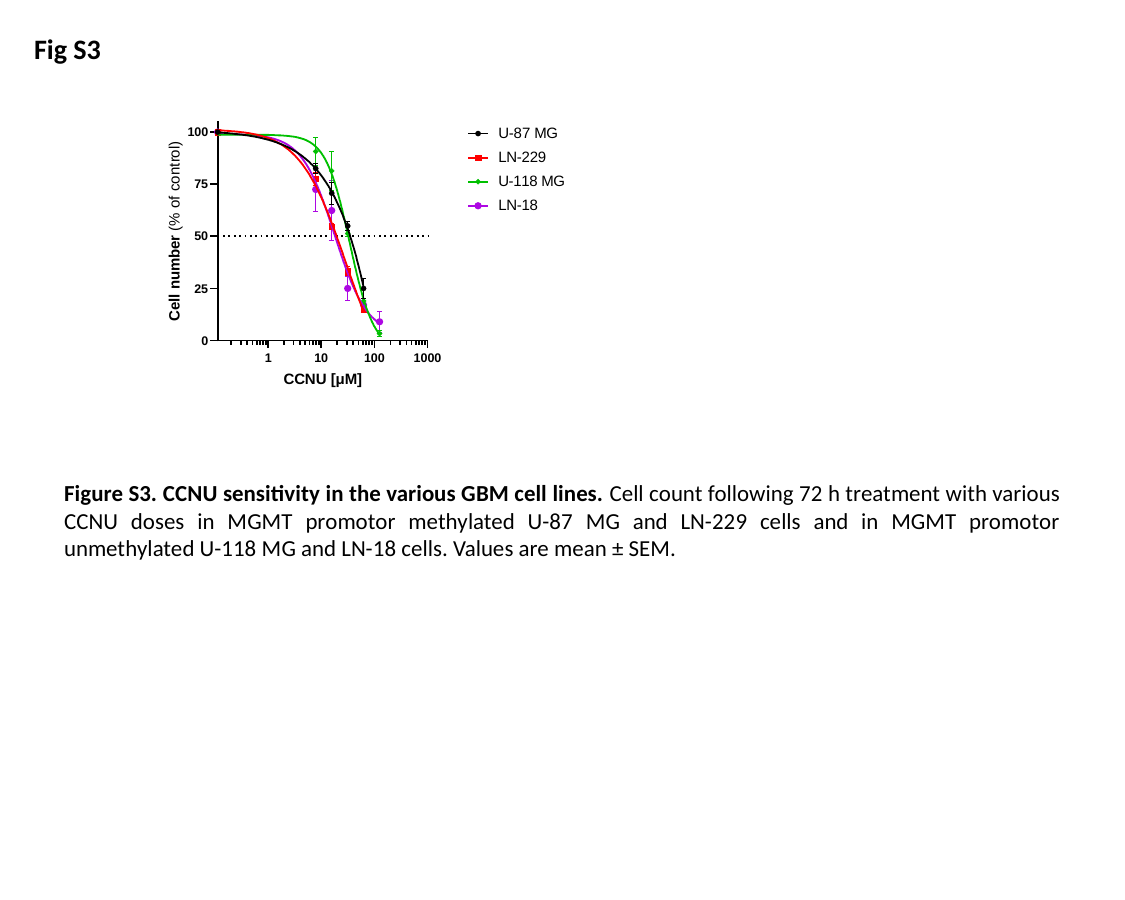

Fig S3
Figure S3. CCNU sensitivity in the various GBM cell lines. Cell count following 72 h treatment with various CCNU doses in MGMT promotor methylated U-87 MG and LN-229 cells and in MGMT promotor unmethylated U-118 MG and LN-18 cells. Values are mean ± SEM.
